# Evaluation of the effect of previous endometriosis surgery on clinical and surgical outcomes of subsequent endometriosis surgery

**DOI:** 10.1007/s00404-023-07193-4

**Published:** 2023-08-28

**Authors:** Fokkedien H. M. P. Tummers, Sophie I. Peltenburg, Jeroen Metzemaekers, Frank Willem Jansen, Mathijs D. Blikkendaal

**Affiliations:** 1 Department of Gynecology, Leiden University Medical Center, Leiden, The Netherlands; 2 Department of Biomechanical Engineering, Delft University of Technology, Delft, The Netherlands; 3 Endometriosis Center, Haaglanden Medical Center, The Hague, The Netherlands; 4 Present Address: Nederlandse Endometriose Kliniek, Reinier de Graaf Hospital, Delft, The Netherlands

**Keywords:** Endometriosis, Repeat surgery, Complications, Patient journey, Recurrence

## Abstract

**Purpose:**

Patients often undergo repeat surgery for endometriosis, due to recurrent or residual disease. Previous surgery is often considered a risk factor for worse surgical outcome. However, data are scarce concerning the influence of subsequent endometriosis surgery.

**Methods:**

A retrospective study in a centre of expertise for endometriosis was conducted. All endometriosis subtypes and intra-operative steps were included. Detailed information regarding surgical history of patients was collected. Surgical time, intra-operative steps and major post-operative complications were obtained as outcome measures.

**Results:**

595 patients were included, of which 45.9% had previous endometriosis surgery. 7.9% had major post-operative complications and 4.4% intra-operative complications. The patient journey showed a median of 3 years between previous endometriosis surgeries. Each previous therapeutic laparotomic surgery resulted on average in 13 additional minutes (*p* = 0.013) of surgical time. Additionally, it resulted in more frequent performance of adhesiolysis (OR 2.96, *p* < 0.001) and in a higher risk for intra-operative complications (OR 1.81, *p* = 0.045), however no higher risk for major post-operative complications (OR 1.29, *p* = 0.418). Previous therapeutic laparoscopic endometriosis surgery, laparotomic and laparoscopic non-endometriosis surgery showed no association with surgical outcomes. Regardless of previous surgery, disc and segmental bowel resection showed a higher risk for major post-operative complications (OR 3.64, *p* = 0.017 respectively OR 3.50, *p* < 0.001).

**Conclusion:**

Previous therapeutic laparotomic endometriosis surgery shows an association with longer surgical time, the need to perform adhesiolysis, and more intra-operative complications in the subsequent surgery for endometriosis. However, in a centre of expertise with experienced surgeons, no increased risk of major post-operative complications was observed.

## What does this study add to the clinical work?

**Table:** 

Patients with previous laparotomic therapeutic endometriosis surgery need to be considered to be at risk for intra-operative complications. However, in experienced hands, this does not lead to increased post-operative complications. Patients with previous laparoscopic endometriosis surgery should not be considered at risk for more intra- or postoperative complications.	

## Introduction

Around 10% of all fertile women is affected by endometriosis [1]. Endometriosis consists of a heterogeneous group of lesions with three subtypes: peritoneal endometriosis (PE), ovarian endometriosis (OMA) and deep endometriosis (DE). Endometriosis can cause high morbidity and could negatively impact quality of life, for which surgical excision is an important treatment modality. However, depending on the length of follow-up and definition, recurrence of endometriosis can be regarded common with 2–43%, within 2–4 years and is higher if pain is the definition rather than surgical findings [2]. Up to 35% of the patients need repeated surgery, with a follow-up up to 80 months [2]. The need for repeat surgery could be due to recurrence of disease or residual disease [2]. Endometriosis could recur spontaneously or if previous surgery was performed (microscopically) irradically [2, 3]. Additionally, residual disease might be the reason for repeat surgery if disease was purposely left in situ, for example, if risks of complications outweigh the symptoms, or to preserve fertility. Still, many endometriosis patients eventually undergo multiple surgical procedures, therefore being an important phenomenon in the surgical patient journey of endometriosis patients.

Outside the endometriosis surgical field, the effect of multiple abdominal surgeries is researched for adverse outcomes. Although some studies show no effect on complications [4–6], most studies showed it to be a risk factor for adhesions [7, 8], longer surgical time [7], intraoperative [9], post-operative complications [10–14] and conversions [15], where for the latter three, adhesions are regarded as major cause [16, 17]. Most of the studies do not distinguish between laparotomy and laparoscopy, whilst other studies specifically review previous laparotomy. Although research focussed on the effect of previous laparoscopy is scarce, post-operative adhesion formation could also occur after laparoscopy [18, 19].

It is uncertain if abovementioned risks are also applicable to endometriosis surgery due to limited evidence. Poupon et al. and Kumakiri et al. showed previous endometriosis surgery to be a risk factor for complications in subsequent endometriosis surgery. However, previous surgery was included as the presence of any number of previous surgical procedures. Additionally, for Poupon et al., the modality (laparoscopy or laparotomy) was not defined [20, 21].

In line with literature of non-endometriosis surgery, our hypothesis is that extensive previous endometriosis surgeries result in worse surgical outcomes. To optimise counselling of the patient and pre-operative planning, more detailed information regarding the risk of previous endometriosis surgeries and surgical outcomes is necessary. Therefore, the aim of this study is to identify the detailed effect of previous endometriosis surgeries on surgical outcomes.

## Methods

A retrospective mono-centre cohort study was conducted in a Dutch centre of expertise for endometriosis. All women who underwent therapeutic endometriosis surgery between January 2019 and February 2021 were included. To select these patients, the Business Intelligence (BI) department of the hospital was asked to provide a list of patients who were scheduled for a therapeutic laparoscopy for endometriosis based on electronic patient data. All subtypes of endometriosis (PE, OMA and DE) and all intra-operative steps (i.e. bowel surgery, bladder surgery, and ureter surgery) were included. Patients with and without previous endometriosis surgery were included, so that the latter group could act as controls. Patients were excluded if no endometriosis was detected or treated during surgery. Surgery was performed by experienced gynaecologists and surgeons, with both more than 10 years of expertise. In the researched centre, over 350 endometriosis surgeries are performed annually. All women had a follow-up of 6 weeks after surgery.

Data was extracted from medical records, including surgical reports. Baseline characteristics, including patient clinical and demographic characteristics, were assessed. The following parameters were included: gravidity, parity, menopausal status, smoking habits, previous abdominopelvic surgery. In case of previous surgeries, the total number of surgeries, and for every surgery the year of surgery, indication (endometriosis-related or non-endometriosis-related such as i.e. appendectomy or cholecystectomy), and modality (laparoscopic vs. laparotomic and diagnostic vs. therapeutic) was obtained. Specifically for endometriosis surgeries, the following additional parameters were obtained: performed in a centre of expertise (defined by the Dutch Endometriosis Foundation), type of treated endometriosis (only PE, OMA (without DE) or DE) and, if available, the rASRM and Enzian scores.

The endometriosis surgery in the researched centre was considered the surgery of interest (index surgery). In case of multiple surgeries in this centre, the last surgery which met the inclusion criteria was considered as the index surgery. These data were assessed from the surgery reports. For this surgery, type of endometriosis treated, rASRM and Enzian scores, intraoperative steps, intra-operative complications, postoperative major complications and surgical time were reported. Missing rASRM scores were reported as missing data. Missing Enzian scores were separately scored by FHMPT and SIP based on surgical reports, and if those were inconclusive ultrasonography and MRI reports were used. When no consensus was achieved, a gynaecologist (MDB) noted the final Enzian score. Intraoperative steps noted were intestinal procedures (appendectomy, bowel shave, disc and segmental resections), urological procedures (partial bladder resection, ureter re-implantation and ureterolysis), hysterectomy and adhesiolysis. The removal of adhesions was only performed if it was required to visualise the operative field to allow safe excision of the endometriosis and was only considered adhesiolysis if a considerable amount of dissection was necessary (e.g. stump removal of filmy adhesions was not considered adhesiolysis).

Intraoperative complications were categorised into vascular, visceral or urinary tract injury or other complications. Postoperative complications were recorded in accordance with the Clavien–Dindo (CD) classification [22], with CD IIIa-V considered as major complications [23]. The onset of a complication had to be within 6 weeks after surgery. However, if a major complication was evidently inherent to the intraoperative steps, i.e. anastomotic leakage after a segmental resection after 8 weeks, this complication was included in the analysis.

### Statistical analysis

IBM SPSS Version 27 was used for statistical analysis. Mean and standard deviation (SD) were used to assess normal distributed descriptive data, and median and interquartile range (IQR) for non-normal distributed data. Independent *t*-tests were used to compare continuous equally distributed variables, Mann–Whitney *U* tests for unequally distributed variables, chi-square or Fisher’s exact test for categorical data. For simple regression analysis, the following variables were selected, based on the main research question and relevant intra-operative procedures of the index surgery affecting outcome measures: the number of therapeutic laparoscopic and laparotomic endometriosis surgeries, the number of therapeutic non-endometriosis laparoscopic and laparotomic surgeries, hysterectomy, bowel surgery (combined and separated between shaving, disc resection, and segmental resection), adhesiolysis and ureterolysis. Diagnostic previous surgeries were excluded from the analysis. For multiple regression analysis, variables from the simple regression analysis with *p* < 0.10 were included. *p* < 0.05 was considered statistically significant.

### Ethics approval

An exemption from review was provided by the Medical Ethical Committee Leiden Delft The Hague (METC LDD, G20.195).

## Results

### Patient characteristics

**Fig. 1:**
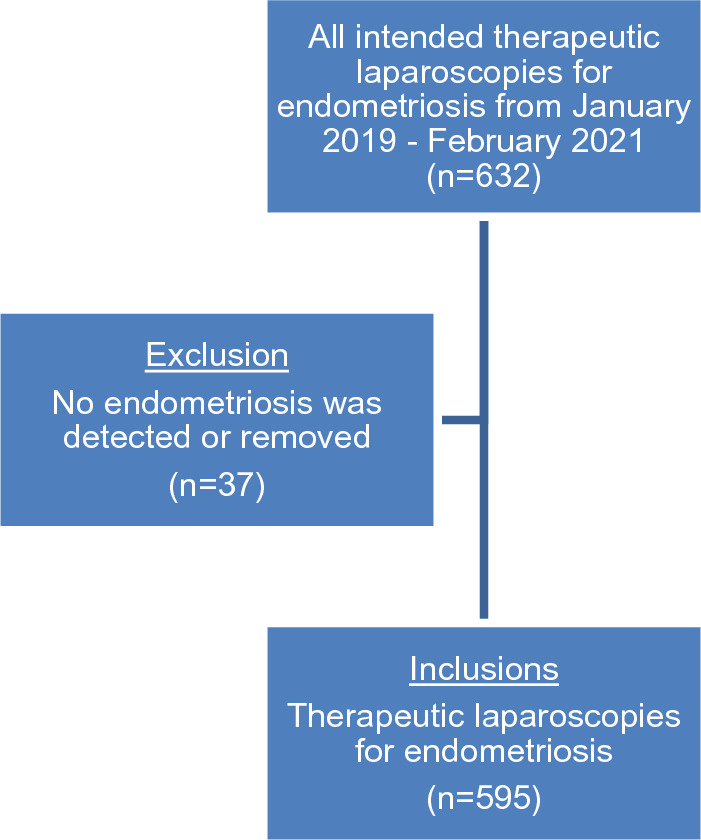
Flowchart with inclusion and exclusion of patients

The BI list included 632 patients. After checking the surgical reports, 37 patients were excluded as no endometriosis was detected or removed during surgery. 595 patients were eligible for inclusion (see Fig. 1). Table 1 shows the patient characteristics. 45.9% (*n* = 273) of all patients had endometriosis surgery in the past, and 14.5% of them had a previous hysterectomy. The age between the patients with and without previous endometriosis surgery differed significantly (37.5 vs. 35.1 years, *p* < 0.001). The percentage patients with BMI > 30 was higher in the previous endometriosis surgery group (18% versus 11%, *p* < 0.013). Patients who had endometriosis surgery before, were younger during the first surgery (30.9 vs. 35.1 years, *p* < 0.001).

**Table 1:** Patient characteristics

Characteristics	All women	No previous endometriosis surgery	Previous endometriosis surgery	*p*-value
	*(N* = *595)*	*(N* = *322)*	*(N* = *273)*	
Age at surgery (years), *mean (SD)*	36.2 (7.09)	35.1 (7.25)	37.5 (6.69)	< 0.001
BMI (kg/m^2^)*, N (%)*				
< 18	13 (2.2)	9 (2.8)	4 (1.5)	NS
18–25	322 (54.1)	185 (57.5)	137 (50.2)	NS
25–30	176 (29.6)	93 (28.9)	83 (30.4)	NS
> 30	84 (14.1)	35 (10.9)	49 (17.9)	0.013
Smoking habits, *N (%)*				
Currently smoker	120 (20.2)	62 (19.4)	58 (21.7)	NS
Never or ex -smoker	475 (79.8)	257 (80.6)	209 (78.3)	NS
Gravidity*, median (IQR)*	0 (0–2)	0 (0–2)	1 (0–2)	NS
Parity*, median (IQR)*	0 (0–1)	0 (0–1)	0 (0–1)	NS
Menopausal state*, N (%)*				
Premenopausal	588 (98.8)	319 (99.1)	168 (98.5)	NS
Postmenopausal	7 (1.2)	3 (0.9)	4 (1.5)	NS
Referred by,* N (%)*				
GP or NGS	165 (27.7)	126 (39.1)	39 (14.3)	< 0.001
Gynaecologist	360 (60.5)	192 (59.6)	168 (61.5)	NS
Patient from own expertise centre	70 (11.8)	4 (1.2)	66 (24.2)	< 0.001
Previous surgery [median (IQR)]	1 (1–2)	0 (0–1)	2 (1–3)	< 0.001
Previous non-endometriosis surgery	0 (0–1)	0 (0–1)	0 (0–1)	0.474
Previous endometriosis surgery	0 (0–1)	0 (0–0)	1 (1–2)	< 0.001
Age at first endometriosis surgery^a ^(years)*, mean (SD)*	33.2 (7.18)	35.1 (7.25)	30.9 (6.42)	< 0.001
Years since last endometriosis surgery, *median (IQR)*	3 (1–7)	–	3 (1–7)	N/A

*NS* no statistical significance, *N/A* not applicable, *IQR* inter-quartile range, *SD* standard deviation, *GP* general practitioner, *NGS* non-gynecological specialist

^a^For patients without previous endometriosis surgery, the age at index surgery is used

### Surgical history

**Fig. 2:**
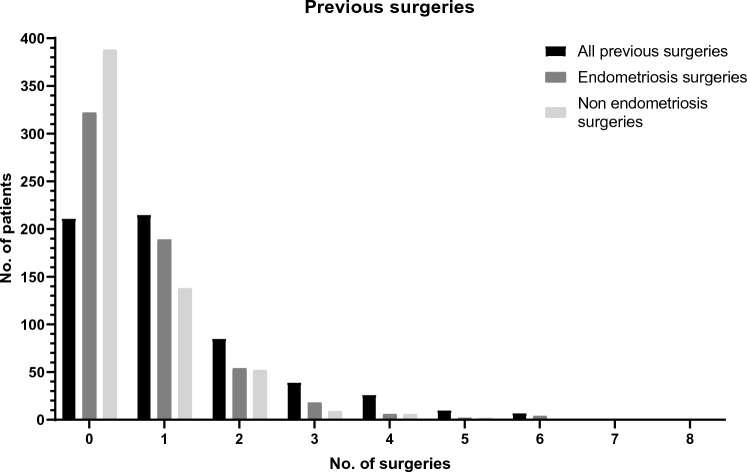
Number of previous all previous surgeries. All previous surgeries are also divided between endometriosis and non-endometriosis surgeries. *x*-axis shows number of surgeries, *y*-axis shows number of patients

**Fig. 3:**
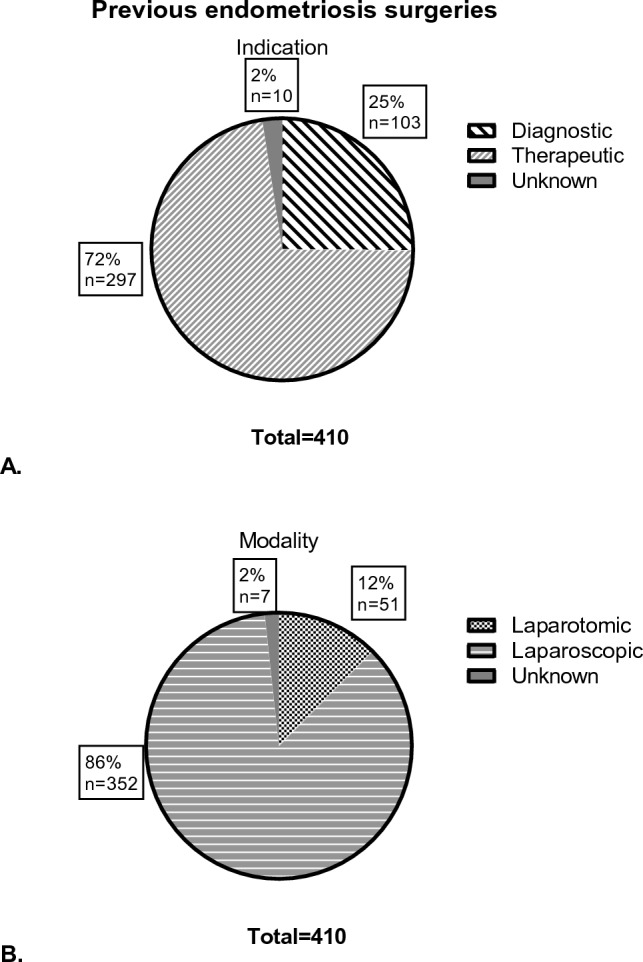
Previous endometriosis surgeries categorised according to indication (**A**) and modality (**B**)

273 patients underwent previous endometriosis surgery, with 410 procedures in total. 11.0% of those patients had 3 or more previous endometriosis surgeries. Figure 2 shows the distribution of the number of previous surgeries. Figure 3A and B shows the distribution of endometriosis surgeries based on modality and indication. Of all non-endometriosis surgeries (*n* = 296), 44% was performed laparoscopic, 40% was laparotomic, and for 16%, it was unknown. Furthermore, 86% was therapeutic and 12% was diagnostic. PE was removed in 21% of the previous surgeries, OMA in 40% and DE in 20%, with sometimes multiple types being removed during the same surgery. If a hysterectomy was previously performed, that surgery was considered as ‘endometriosis surgery’. Most of the hysterectomies were combined with the removal of PE, OMA or DE. In surgeries without resection of other endometriosis, most of those patients had endometriosis surgery for PE, OMA or DE before, and it was therefore assumed that the need to remove the uterus in patients with endometriosis most likely would have been related to symptoms linked to endometriosis.

**Fig. 4:**
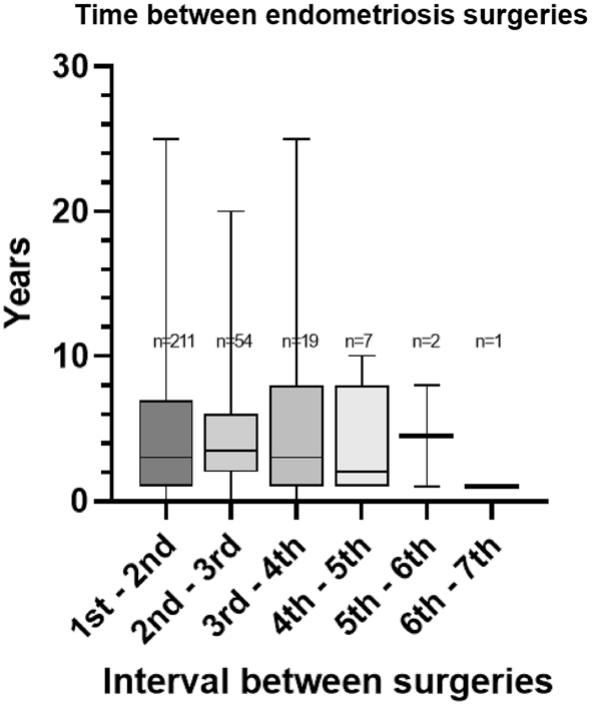
Time in years between endometriosis surgeries, determined per patient. Box shows inter-quartile range and median, whiskers show minimum and maximum

Of all previous endometriosis surgeries, 32% was performed in a centre of expertise, including 24% performed in the same centre as the index surgery. Also for the patients with 3 or more previous endometriosis surgeries, 37% of those surgeries were performed in these centres, showing no difference with the total cohort. Figure 4 shows the time path of previous endometriosis surgeries. The median time between surgeries is shown to be relatively equal, with the last 2 intervals including, respectively, only 2 and 1 patient.

### Endometriosis classification of index surgery

The endometriosis classifications (rASRM and ENZIAN) did not differ significantly between the patients with and without previous endometriosis surgery (Table 2). The results show a diverse patient population, including 33% of the patients with bowel endometriosis.

**Table 2:** Endometriosis classification during index surgery

Characteristics	All women	No previous endometriosis surgery	Previous endometriosis surgery	*p*-value
	*(N* = *595)*	*(N* = *322)*	*(N* = *273)*	
rASRM stage, *N (%)*				
Stage 1	171 (28.7)	100 (31.1)	71 (26.0)	NS
Stage 2	98 (16.5)	60 (18.6)	38 (13.9)	NS
Stage 3	106 (17.8)	54 (16.8)	52 (19.0)	NS
Stage 4	159 (26.7)	83 (25.8)	76 (27.8)	NS
Unknown	61 (10.3)	25 (7.7)	36 (13.2)	NS
Enzian stage,* N (%)*				
*A (vagina)*				
< 1 cm	12 (2.0)	6 (1.9)	6 (2.2)	NS
1–3 cm	40 (6.7)	21 (6.5)	19 (7.0)	NS
> 3 cm	78 (13.1)	41 (12.7)	37 (13.6)	NS
No endometriosis in this compartment	465 (78.2)	254 (78.9)	211 (77.3)	NS
*B (ligaments)*				
< 1 cm	60 (10.1)	35 (10.9)	25 (9.2)	NS
1–3 cm	163 (27.4)	90 (28.0)	73 (26.7)	NS
> 3 cm	119 (20.0)	64 (19.9)	55 (20.1)	NS
No endometriosis in this compartment	252 (42.4)	132 (41.1)	120 (44.0)	NS
*C (rectum)*				
< 1 cm	20 (3.4)	8 (2.5)	12 (4.4)	NS
1–3 cm	60 (10.1)	33 (10.3)	27 (9.9)	NS
> 3 cm	114 (19.2)	61 (19.0)	53 (19.4)	NS
No endometriosis in this compartment	400 (67.2)	219 (68.2)	181 (66.3)	NS
*F (far)*				
FA (adenomyosis)	362 (60.8)	204 (63.4)	158 (57.9)	NS
FB (bladder)	76 (12.8)	41 (12.7)	35 (12.8)	NS
FU (ureter)	102 (17.1)	53 (16.5)	49 (17.9)	NS
FI (intestinal)	126 (21.1)	58 (18.0)	68 (24.9)	NS
FO (other)	36 (6.1)	25 (7.8)	11 (4.0)	NS
No deep endometriosis present	56 (9.4)	26 (8.1)	30 (11.0)	NS

*NS* no statistical significance

### Surgical characteristics

Table 3 shows the surgical characteristics of the index surgery. All procedures started laparoscopically, with 1 strategic conversion to laparotomy in a patient without previous surgery for endometriosis. In almost 70% of the surgeries, DE was removed. Several intra-operative procedures were performed, including segmental bowel resection in 20.8% of the patients. Adhesiolysis was performed more often in the group with previous endometriosis surgery (30.0% vs. 18.8%, *p* = 0.001). Simple regression analysis showed that previous laparotomic and laparoscopic endometriosis surgeries were both significantly associated with the need to perform adhesiolysis (OR 2.96, *p* < 0.001 and OR 1.29, *p* = 0.040, respectively). Non-endometriosis therapeutic surgery (both laparotomic and laparoscopic) was not associated with adhesiolysis (OR 1.19, *p* = 0.316 and OR 1.36, *p* = 0.117, respectively).

**Table 3:** Surgical characteristics of index surgery

Characteristics	All women	No previous endometriosis surgery	Previous endometriosis surgery	*p*-value
	*(N* = *595)*	*(N* = *322)*	*(N* = *273)*	
Surgical time, *median (IQR)*	90 (50–120)NS	90 (50–120)	90 (50–130)	NS
Surgical approach,* N (%)*				
Laparoscopy	594 (99.8)	321 (99.7)	273 (100)	NS
Laparotomy	–	–	–	N/A
Conversion, strategic	1 (0.2)	1 (0.3)	0 (0)	NS
Type of endometriosis treated, *N (%)*^a^				
Peritoneal	308 (51.8)	170 (52.8)	138 (50.5)	NS
Endometrioma	218 (36.6)	108 (33.5)	110 (40.3)	NS
Deep	416 (69.9)	226 (70.2)	190 (69.6)	NS
Hysterectomy	210 (35.3)	116 (36.0)	94 (34.4)	NS
Most severe endometriosis type treated^b^				NS
Peritoneal	68 (11.4)	33 (10.2)	35 (12.8)	
Endometrioma	66 (11.1)	31 (9.6)	35 (12.8)	
Deep	416 (69.9)	226 (70.2)	190 (69.6)	
Intraoperative procedures^c^,* N (%)*				
Hysterectomy	210 (35.3)	116 (36.0)	94 (34.4)	NS
*Indication adenomyosis*	*184 (30.1)*	*103 (32.0)*	*81 (29.7)*	NS
Appendectomy	48 (8.1)	26 (8.1)	22 (8.1)	NS
Bowel shave	58 (9.7)	25 (7.8)	33 (12.1)	NS
Bowel disc resection	26 (4.4)	16 (5.0)	10 (3.7)	NS
Bowel segment resection	124 (20.8)	67 (20.8)	57 (20.9)	NS
Ureter re-implantation	4 (0.7)	4 (1.2)	0 (0)	NS
Partial bladder resection	13 (2.2)	6 (1.9)	7 (2.6)	NS
Ureterolysis	183 (30.8)	99 (30.7)	84 (30.8)	NS
Adhesiolysis	141 (23.7)	59 (18.3)	82 (30.0)	0.001

*N/A* not applicable, *NS* no statistical significance

^a^More than 1 type of endometriosis could be treated during index surgery

^b^Peritoneal endometriosis was considered least severe and deep endometriosis most severe type

^c^Patients could have had more than 1 intra-operative procedure

### Surgical time

The median surgical time was 90 min (IQR 50–120 min). Previous therapeutic laparotomic endometriosis surgery showed a significant association with increased surgical time (on average requiring 13 min extra surgical time for each previous surgery (*p* = 0.012)), whilst therapeutic laparoscopic endometriosis and laparoscopic and laparotomic non-endometriosis surgery showed no significant association (*p* = 0.921, *p* = 0.356 and *p* = 0.593 respectively). Additionally, performing adhesiolysis required on average 20 min additional surgical time (*p* < 0.001), hysterectomy 31 min (*p* < 0.001), and ureterolysis 27 min (*p* < 0.001). Performing bowel surgery also added significantly to surgical time with 42 min for shaving (*p* = 0.000), 48 min for disc resection and 82 min for segmental resection (both *p* < 0.001).

### Intra-operative complications

29 intraoperative complications were reported in 26 patients (4.4%), of which most were visceral complications (Table 4). 6 patients had visceral injury after adhesiolysis. More intra-operative complications were observed in the group with previous endometriosis surgery (6.2% vs 2.8%, *p* = 0.046), mostly due to more urinary tract injury in this group.

**Table 4:** Number of patients with complications of index surgery

	All women	No previous endometriosis surgery	Previous endometriosis surgery	*p*-value
	*(N* = *595)*	*(N* = *322)*	*(N* = *273)*	
Intraoperative complications^a^ (*N* = 29)*, N (%)*	26 (4.4)	9 (2.8)	17 (6.2)	0.046
Vascular	4 (0.7)	2 (0.6)	2 (0.7)	NS
Visceral	16 (2.7)	6 (1.9)	10 (3.7)	NS
Urinary tract	4 (0.7)	0 (0)	4 (1.5)	0.044
Other	2 (0.3)	1 (0.3)	1 (0.4)	NS
Major post-operative complications^b^ (*N* = 53)*, N (%)*				
Active bleeding	4 (0.7)	1 (0.3)	3 (1.1)	NS
Rectovaginal fistula	4 (0.7)	3 (0.9)	1 (0.4)	NS
Wound infection	1 (0.2)	0 (0)	1 (0.4)	NS
Urinary infection	1 (0.2)	0 (0)	1 (0.4)	NS
Pelvic abscess	3 (0.5)	1 (0.3)	2 (0.7)	NS
Urethral injury	5 (0.8)	2 (0.6)	3 (1.1)	NS
Vaginal dehiscence	6 (1.0)	4 (1.2)	2 (0.7)	NS
Anastomotic leakage	11 (1.8)	8 (2.5)	3 (1.1)	NS
Bowel injury	2 (0.3)	0 (0)	2 (0.7)	NS
Bladder injury	1 (0.2)	0 (0)	1 (0.4)	NS
Other	14 (2.4)	8 (2.5)	6 (2.2)	NS
Major post-operative complications Clavien–Dindo score^c^*, N (%)*				
Grade IIIa	1 (0.2)	0 (0)	1 (0.4)	NS
Grade IIIb	42 (7.1)	23 (7.1)	19 (7.0)	NS
Grade IVa	2 (0.3)	1 (0.3)	1 (0.4)	NS
Grade IVb	3 (0.5)	1 (0.3)	2 (0.7)	NS

Patients could have had more than one intra- or post-operative complication

NS no statistical significance

^a^1 patient had 2 visceral intra-operative complications and 1 patient had 3 visceral intra-operative complications

^b^1 patient had 2 Other complications

^c^5 patients had 2 grade 3b complications

Simple regression showed an association of previous therapeutic laparotomic endometriosis surgery (OR 2.12, *p* = 0.008) and adhesiolysis (OR 2.48, *p* = 0.027) with intra-operative complications. Therapeutic laparoscopic endometriosis surgery (OR 1.11, *p* = 0.694), both therapeutic laparotomic (OR 0.67, *p* = 0.437) and laparoscopic non-endometriosis surgeries (OR 0.75, *p* = 0.607) and all other intra-operative steps showed no association. In multiple regression analysis, the number of previous therapeutic laparotomic endometriosis surgery was still positively associated (OR 1.81 per surgery, *p* = 0.045). However, this association disappeared for adhesiolysis (OR 2.1, *p* = 0.092). This indicates that the effect of previous surgery on intra-operative complications is not fully explained by adhesiolysis.

### Major post-operative complications

In 41 (6.9%) patients, one major post-operative complication was observed and 6 patients (1.0%) had two major complications (Table 4). None of the previous surgeries showed an association with major post-operative complications (OR 1.29, *p* = 0.418; OR 1.04, *p* = 0.853; OR 0.99, *p* = 0.963, OR 1.46, *p* = 0.140, for, respectively, previous therapeutic laparotomic and laparoscopic endometriosis surgeries, and therapeutic laparotomic and laparoscopic non-endometriosis surgeries). Of the intra-operative procedures, only the performance of disc and segmental resection resulted in a higher risk for complications in multiple regression analysis (OR 3.64, *p* = 0.017, respectively, OR 3.50, *p* < 0.001).

## Discussion

Our study shows that the number of previous therapeutic laparotomic endometriosis surgeries was associated with increased surgical time, need for adhesiolysis and occurrence of intra-operative complications (mostly visceral injury), without resulting in more post-operative complications. The number of previous therapeutic laparoscopic endometriosis surgery and both therapeutic laparotomic and laparoscopic non-endometriosis surgery was not associated with any of the outcome measures. Regardless of previous surgery, bowel surgery (i.e. disc and segmental resection) was the most important contributor to post-operative complications.

Two studies researched the effect of previous endometriosis surgeries on complications, although the surgical history was described in little detail. In line with Kumakiri [21], our study showed an association between previous laparotomic endometriosis surgery and increased intraoperative complications. Contrary to their implication that adhesiolysis plays an important role in this effect, our data show that previous surgery also has an effect on intra-operative complications independent from adhesiolysis, indicating an additional effect of previous surgery other than the formation of adhesions. This is supported by the pathophysiology of endometriosis itself, which indicates chronic inflammation and consequently pelvic adhesion formation [24]. Poupon et al. showed an association of previous endometriosis surgery (laparoscopy or laparotomy was not defined) with more post-operative complications [20], in contrary to our study. They only included patients without bowel involvement, indicating a population with less severe endometriosis. This could indicate that in a population with less severe endometriosis and therefore less risky intra-operative procedures, previous surgery might play a more important role as a risk factor. Also, in our cohort, surgical history was described and analysed in more detail which might explain different results, as we described the previous surgeries on a numerous scale instead of dichotomous outcome.

Additionally, in our cohort, performing adhesiolysis did not result in a higher risk for post-operative complications. This could be attributed to the fact that the researched centre is high-volume with experienced gynaecologists, and high-volume hospitals result in less complications [25–30]. Therefore, we cannot state that our results are generalizable to gynaecologists with less experience. However, as our study shows that endometriosis surgery nowadays is almost solely performed laparoscopically, the risk for adhesiolysis and intra-operative complications might decline in the future.

The major complication rate in our study (7.9%) might be considered high compared to some other studies, though no difference was observed between patients with and without previous endometriosis surgery. Kondo et al. [31] show 4.6% major complication rate, Nicolaus et al. 1.7% [32]. However, segmental bowel resection was performed in only 4.4% and 5.5% of their populations respectively, compared to 20.8% in our population. As our study indicates segmental resection as the most important contributor to post-operative complication, that could explain our higher complication rate. Lermann et al. showed 3.7% major complications, but they excluded bowel surgery [33]. Compared to the study of Hera-Lazarro, showing major complications in 17.4% of the patients, whilst performing bowel resection in 21.7% of the patients, our complication rate is lower [34]. Although no additional risk is seen after multiple surgeries, it would certainly be the best for patients to undergo as few surgeries as possible due to the present risk of complications. However, in some cases, multiple surgeries could be preferable (e.g. after a shared decision performing no complete resection of nodules if there are no symptoms or no hysterectomy to preserve fertility). As our study shows no additional risk due to previous endometriosis surgeries, this approach seems to be valid, and could provide important information to the counselling of patients.

Our data show valuable information for patient counselling as it shows which patients are at risk. Patients with previous therapeutic laparotomic endometriosis surgery are at risk of intra-operative complications. However, our study also shows that in experienced hands, this does not lead to more post-operative complications. It also shows that patients with multiple previous laparoscopically endometriosis surgeries are not at risk for more complications, both intra- and postoperatively. Additionally, our study also gives information regarding the patient journey, which could help patients with more realistic expectations. Already 45.9% of the population had undergone previous endometriosis surgery, which indicated a substantial risk for repeat surgery, with overall 1 in 20 patients that had undergone 3 or more endometriosis surgeries. This is slightly lower than in the centre of expertise studied by Agarwal et al., with 60% of their cohort with previous endometriosis surgery [35]. In line with literature, our research showed a relatively equal time of approximately 3 years between concurrent endometriosis surgeries [36, 37], which adds to the expectations of patients. As almost a third of all previous surgeries is already performed in a centre of centre of expertise, and also the patients with 3 or more previous surgeries underwent a third of their previous surgeries in a centre of expertise, less experience does not per se lead to the need for repeat surgery. However, as no information was available regarding the need for repeat surgery (recurrent or residual disease), this conclusion should be taken with caution. Our data also show valuable information for pre-operative planning. Next to the fact that surgeons could anticipate on more adhesions after previous laparotomic endometriosis surgeries, more specific time planning could be made with our results. Frequently performed intra-operative steps, e.g. ureterolysis and bowel surgery are provided with their mean corresponding additional surgical time.

Some limitations need to be mentioned. As this is a retrospective study, not all detailed information regarding previous surgeries was available. However, compared to the scarce already available evidence, even with this limitation, the results of this study are of added clinical value. Additionally, the year of surgery was not available for each surgery, and therefore the time to recurrent surgeries was not available for all patients. A prospective study would result in more extensive and more precise patient journey. Such a prospective study could elaborate more on the reasons for repeat surgeries, as detailed intra-operative information would be available, and choices that were made regarding the intra-operative steps. Next to that, also patients without the need for recurrent surgeries would be identified, as we now miss patients with long-term follow-up without the need for repeat surgery. This would result in a more complete insight into the patient journey within the endometriosis population.

## Conclusion

In this study, performed in a centre of expertise for endometriosis, it is shown that the number of previous therapeutic laparotomic endometriosis surgery increases surgical time (13 min per previous surgery), the need to perform adhesiolysis (OR 2.96) and intra-operative complications (OR 1.81). However, no effect was observed on major post-operative complications. Additionally, previous therapeutic laparoscopic endometriosis surgery and both laparoscopic and laparotomic non-endometriosis surgeries showed no association with all outcome measures. Therefore, patients with previous laparotomic therapeutic endometriosis surgery need to be considered to be at risk for intra-operative complications. However, in experienced hands, this does not lead to a higher risk for post-operative complications. Repeat endometriosis surgery showed to be a common phenomenon, as 45.9% of our population had previous one of more endometriosis surgeries, with a median time of 3 years between surgeries.

## Data Availability

Not applicable.
